# Is fluorine-18 fluorodeoxyglucose positron emission tomography useful for the thyroid nodules with indeterminate fine needle aspiration biopsy? a meta-analysis of the literature

**DOI:** 10.1186/1916-0216-42-38

**Published:** 2013-09-01

**Authors:** Ningjian Wang, Hualing Zhai, Yingli Lu

**Affiliations:** 1 Department of Endocrinology and Metabolism, Shanghai Ninth People’s Hospital, Affiliated to Shanghai Jiaotong University School of Medicine, No. 639, Zhizaoju Road, Shanghai 200011, China

**Keywords:** Fluorine-18 fluorodeoxyglucose, Positron emission tomography, Meta-analysis, Thyroid nodule, Fine needle aspiration biopsy, Indeterminate

## Abstract

**Background:**

The indeterminate fine needle aspiration biopsy (FNAB) results present a clinical dilemma for physicians. The aim of this study was to evaluate the diagnostic accuracy of fluorine-18 fluorodeoxyglucose positron emission tomography (^18^ F-FDG PET) in the detection of these indeterminate lesions.

**Methods:**

Seven studies (involving a total of 267 patients) published before November 2012 were reviewed. Systematic methods were used to identify, select, and evaluate the methodological quality of the studies as well as to summarize the overall findings of sensitivity and specificity.

**Results:**

A total number of 70 patients were confirmed to have malignant lesions, with a cancer prevalence of 26.2% (70/267; ranging from 19.6% to 40.0% in these studies). The pooled sensitivity and specificity of PET or PET/CT for the detection of cancer was 89.0% (95% CI: 79.0% ~ 95.0%) and 55.0% (95% CI: 48.0% ~ 62.0%), respectively. There was no evidence of threshold effects or publication bias. The area [±standard error (±SE)] under the symmetrical sROC curve was 0.7207 ± 0.1041. Although SUVmax was higher in malignant lesions (*P* < 0.01), there was still a great overlap. The best cut-off value of SUVmax for differentiation was 2.05; but with a high sensitivity of 89.8% and low specificity of 42.0%.

**Conclusion:**

F-FDG PET or PET/CT showed a high sensitivity in detecting thyroid cancers in patients with indeterminate FNAB results. Further examination was strongly recommended when an FDG-avid lesion was detected.

## Background

Thyroid cancer is the most common endocrine malignancy. It represents approximately 1% of all cancers, corresponding to an incidence of up to 56, 460 new cases per year in the United States, with increasing incidence over the last decades [1]. Early identification and diagnosis is important in appropriate treatment of thyroid cancer, as delays in the diagnosis are associated with increased mortality [2]. The major diagnostic challenge in the work-up of the large number of patients with thyroid nodules is to select for surgery only those patients with malignant nodules.

Current guidelines recommend performing fine needle aspiration biopsy (FNAB) for nodules with a diameter larger than 5 mm to 20 mm, depending on patient clinical history and presence of suspicious ultrasonographic findings [3]. FNAB is safe, easily performed, without major complications, and cost-effective. However, a number of reports have shown that between 11% and 42% of FNABs of thyroid lesions are reported as indeterminate [4-6]. These cytological indeterminate nodules present a clinical dilemma to the evaluating clinician. Therefore, patients with indeterminate or suspicious FNAB results have to undergo diagnostic hemithyroidectomy to exclude malignancy [7]. Because only 20% to 30% of these nodules are malignant [8], most patients are undergoing unnecessary thyroid surgery with the potential risk of irreversible complications.

Unfortunately, at present, there is no alternative algorithm for a more conservative management of patients with thyroid nodules of indeterminate cytopathology. As a molecular image modality, Fluorine-18 Fluorodeoxyglucose (^18^ F-FDG) PET can detect a wide variety of tumor sites [9]. Several studies have reported the role of PET in assessing cytological indeterminate nodules [10-18]. Nevertheless, the reduced number of patients in these studies and the existence of conflicting or non-conclusion results make it impossible to draw a definite conclusion. The purpose of this study was to meta-analyze published data on the diagnostic performance of ^18^ F-FDG PET or PET/CT in predicting the correct diagnosis of cytological indeterminate thyroid nodules.

## Methods

### Search strategy

This project was approved by the institutional review board at Shanghai Ninth People’s Hospital Affiliated to Shanghai Jiaotong University School of Medicine. Studies were identified in the following electronic databases: PubMed/MEDLINE and Embase databases. The search was updated until November 2012 and no beginning date limit was used. We searched articles by using the key words: “PET”; “PET/CT”; “thyroid nodule”; “thyroid incidentaloma”; “FDG”; “FNA”; “FNAB”; “indeterminate”; “unsuspicious” or “non-conclusion”. Only English-language was considered because the investigators were not familiar with other languages. To expand our search, bibliographies of articles that finally remained after the selection process were screened for potentially suitable references.

### Study selection

Two investigators independently evaluated potential studies for inclusion and subsequently resolved disagreements by discussion. Reviewers of the studies were blinded to journal, author, institutional affiliation, and date of publication. The selection criteria were: (i) adult patients with thyroid nodule with indeterminate FNAB; (ii) definite histological or follow up outcome; (iii) using ^18^ F-FDG PET or PET/CT for further diagnosis; (iv) a study population of at least ten patients and (v) the reported primary data were sufficient to allow calculation of both sensitivity and specificity. When data were presented in more than one article, the article with the largest number of patients or the article with the most details was chosen.

Exclusion criteria were: (i) studies that presented results from a combination of different imaging modalities that could not be differentiated for the assessment of a single test date; (ii) animal studies and (iii) abstracts, reviews, editorials, letters and comments.

### Study quality

Quality appraisal of retrieved full-text articles was all graded independently by 2 investigators for quality and applicability according to the Quality Assessment Tool for Diagnostic Accuracy Studies (QUADAS). This widely used tool consists of 14 items that cover patient spectrum, reference standard, disease progression bias, verification and review bias, clinical review bias, incorporation bias, test execution, study withdrawals, and intermediate results [19,20]. Disagreements were resolved by consensus after a re-evaluation of the references.

### Data analysis

Data were reported according to the guidelines for meta-analysis evaluating diagnostic tests. For each study, the sensitivity, specificity and their 95% confidence intervals (CI) were calculated from the original data. Positive likelihood ratio (LR+), negative likelihood ratio (LR-), and diagnostic odds ratio (DOR) were also reported. To avoid calculation problems by having zero values, 0.5 was added to each cell of the respective contingency table, which is a common method [21].

Because sensitivity and specificity often are related inversely because of the threshold effect, study heterogeneity in these diagnostic test characteristics was observed using a summary receiver operating characteristic (sROC) curve for which the area under the curve (AUC) was calculated.

All meta-analyses (pooling and sROC analysis) were performed using Meta-DiSc (version 1.4; Unit of Clinical Biostatistics, Ramon y Cajal Hospital, Madrid, Spain) [22].

The Mann–Whitney *U* test were used to compare PET findings between benign and malignant lesions. We utilized ROC curve analysis to find the best cut-off value of SUVmax for differentiation. Data was analyzed by SPSS 13.0 software. All analyses were two-sided. A *P* value less than 0.05 was taken to indicate a significant difference.

## Results

### Literature review

From the computer search and after extensive crosschecking of reference lists, 283 abstracts were retrieved. Reviewing titles and abstracts revealed 18 articles potentially eligible for inclusion. Thus, these 18 articles were retrieved in full text version. Screening of the references of these articles did not bring up new articles. Eleven articles were excluded from meta-analysis because: (i) researchers in the articles did not use histopathologic analysis and/or follow-up as the reference standard (n = 6); (ii) researchers in the articles did not report data that could be applied to construct or calculate true-positive, false-positive, true-negative, and/or false-negative results (n = 3); (iii) lesion by lesion analysis only (n = 1) and (iv) no FDG negative results (n = 1). Therefore, 7 studies with 267 patients were finally included in the meta-analysis.

### Study description

The characteristics of these 7 studies were demonstrated in Table 1. The selected studies were performed in Austria [10], Netherland [11], Brazil [12], America [13-15] and France [16]. The age of the patients included in the selected studies ranged from 18 to 84 years, with an average age ranging from 45.3 to 55.1 years. The sex distribution was described in all of the 7 studies, and the men-to-women ratio was 0.21 (46/221).

**Table 1:** The characteristics of these 7 studies

**Study**	**Year**	**Country**	**Design**	**Modality**	**No. of patients**	**Male/Female**	**Age (Mean ± SD)**
Kresnik [10]	2003	Austria	NR	PET	37	8/29	55.1 ± 13.8
de Geus-Oei [11]	2006	Netherlands	Prospective	PET	44	3/41	48.5 ± 13.8
Sebastianes [12]	2007	Brazil	NR	PET	42	4/38	45.3 ± 16.3
Hales [13]	2008	America	Prospective	PET/CT	15	1/14	47.5 ± 14.9
Smith [14]	2008	America	Prospective	PET	23	7/16	50*
Traugott [15]	2010	America	Prospective	PET, PET/CT	51	10/41	49.6 ± 10.6
Deandreis [16]	2012	France	Prospective	PET/CT	55	13/42	50.7 ± 10.5

*NR*  not reported, *SD*  the standard deviation; * The SD of the study was not reported.

Among 7 studies, 4 were evaluated by PET [10-12,14], 2 were evaluated by PET/CT [13,16] and the other 1 was evaluated by either PET or PET/CT [15]. Most of these studies were prospective [11,13-16], and the type of two studies was not specified [10,12].

In all but 1 study [13], all patients had thyroid-stimulating hormone (TSH) levels within the normal range. ^18^ F-FDG PET studies were obtained from all patients at least 2 weeks after FNAB [11,12] while the other 5 studies did not mention this interval [10,13-16].

The inclusion, exclusion criteria and definition of FDG-PET positivity of these 7 studies were shown in Table 2.

**Table 2:** The inclusion, exclusion criteria and definition of FDG-PET positivity of these 7 studies

**Study**	**Year**	**Inclusion criteria**	**Exclusion criteria**	**Definition of FDG-PET positivity**
Kresnik [10]	2003	All patients with TN; hypoechogenic or no uptake on scintigraphy; follicular or Hürthle cell proliferation on FNAB; scheduled for surgery	Autonomous goiter	Focal uptake with SUV > 2
de Geus-Oei [11]	2006	Palpable TN; FNAB: a follicular neoplasm or a Hürthle cell (oncocytic) neoplasm, if they showed atypical papillary cells, or if the sample was repeatedly insufficient; scheduled for hemithyroidectomy	DM; pregnancy	Focal uptake
Sebastianes [12]	2007	FNAB: hypercellular follicular and oxyphilic nodules suggestive of follicular neoplasm and also includes lesions that are suspect but not diagnostic of papillary carcinoma; scheduled for hemithyroidectomy	Uncontrollable DM; other malignancies; pregnancy; abnormal TSH	Focal uptake
Hales [13]	2008	All patients with TN; either follicular or Hürthle cell lesion by FNAB	Pregnancy; breastfeeding; previous neck surgery; >181 kg bodyweight	Focal uptake with SUV >2
Smith [14]	2008	Patients with a preoperative diagnosis of follicular neoplasm by FNAB for whom surgical excision was planned	None	Area under SUV curve > 175.5
Traugott [15]	2010	Adults with TN or dominant TN; palpable or >1 cm on Utrasound; scheduled for surgery; FNAB: follicular lesion, Hürthle cell or oncocytic cell lesion, atypical cytology, abnormal cytology, or suspicious cytology	Previous neck surgery; previous radiotherapy	Focal uptake
Deandreis [16]	2012	Patient age > 18 years; TN with a diameter ≥ 1cm; FNAB: indeterminate follicular lesions	DM; abnormal TSH	Focal uptake

*TN*: thyroid nodule, *DM*: diabetes mellitus.

### Study quality

Among the 7 studies, none of them reported the interval between PET and surgery (QUADAS Item 4). Besides, none of the reviewed articles interpreted the ^18^ F-FDG PET and histology data in combination with other clinical data that would be available in practice (QUADAS Item 12). Only 2 articles described the methodology of reference test in sufficient detail to permit replication (QUADAS Item 9) [14,16], and only 1 article mentioned blinding of the pathologist to the PET findings (QUADAS Item 11) [15].

### Quantitative analysis (Meta-Analysis)

**Figure 1:**
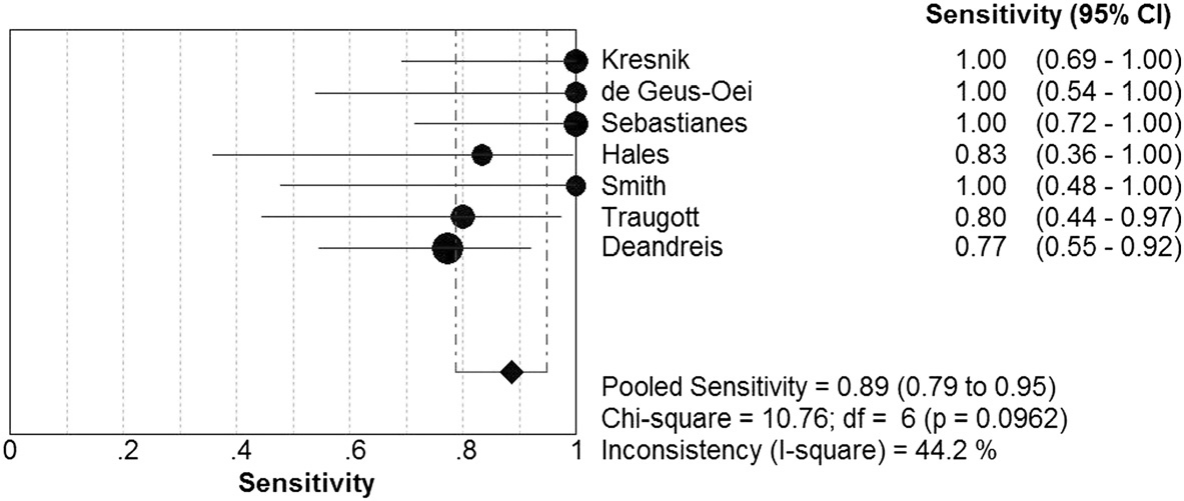
The pooled sensitivity of PET or PET/CT for the detection of cancer.

**Figure 2:**
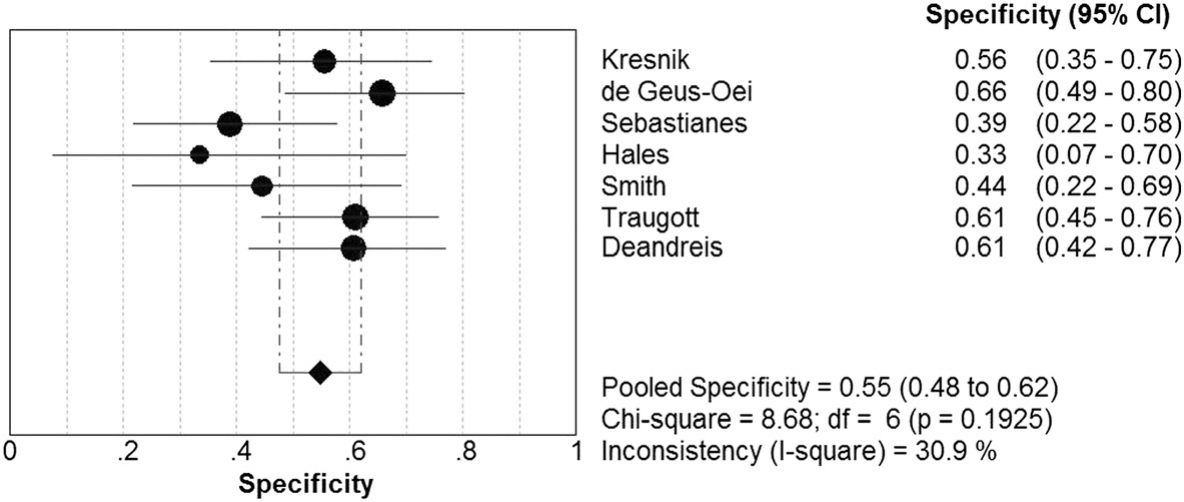
The pooled specificity of PET or PET/CT for the detection of cancer.

7 studies evaluated 267 patients with indeterminate FNAB results. The pooled sensitivity of PET or PET/CT for the detection of thyroid cancer was 89.0% (95% CI: 79.0% ~ 95.0%; I^2^ = 0.442; chi-square test with 6 degrees of freedom [df] = 10.76; *P* = 0.0962). The pooled specificity was 55.0% (95% CI: 48.0% ~ 62.0%; I^2^ = 0.309; chi-square test with 6 df = 8.68; *P* = 0.1925). There was no significant inconsistency among these studies (Figures 1, 2). Therefore, a fixed-effects model was used to calculate these pooled data. The overall LR+, LR- and DOR were 1.87 (95% CI: 1.57 ~ 2.23), 0.24 (95% CI: 0.13 ~ 0.45) and 8.15 (95% CI: 3.89 ~ 17.08), respectively. Besides, the LR+, LR- and DOR for each of the studies were presented in Table 3.

**Table 3:** Reported LR+, LR-, and DOR of these 7 studies

**Study**	**Year**	**TP**	**TN**	**FP**	**FN**	**LR + (95% CI)**	**LR- (95% CI)**	**DOR (95% CI)**
Kresnik [10]	2003	10	15	12	0	2.14 (1.39 ~ 3.29)	0.08 (0.01 ~ 1.26)	26.04 (1.39 ~ 489.26)
de Geus-Oei [11]	2006	6	25	13	0	2.68 (1.66 ~ 4.33)	0.11 (0.01 ~ 1.59)	24.56 (1.28 ~ 469.69)
Sebastianes [12]	2007	11	12	19	0	1.57 (1.16 ~ 2.13)	0.11 (0.01 ~ 1.66)	14.74 (0.80 ~ 273.13)
Hales [13]	2008	5	3	6	1	1.25 (0.70 ~ 2.24)	0.50 (0.07 ~ 3.75)	2.50 (0.19 ~ 32.19)
Smith [14]	2008	5	8	10	0	1.66 (1.04 ~ 2.66)	0.19 (0.01 ~ 1.16)	8.90 (0.43 ~ 184.86)
Traugott [15]	2010	8	25	16	2	2.05 (1.25 ~ 3.35)	0.33 (0.09 ~ 1.16)	6.25 (1.17 ~ 33.26)
Deandreis [16]	2012	17	20	13	5	1.96 (1.21 ~ 3.17)	0.38 (0.17 ~ 0.85)	5.23 (1.55 ~ 17.67)
Pooled results		62	108	89	8	1.87 (1.57 ~ 2.23)	0.24 (0.13 ~ 0.45)	8.15 (3.89 ~ 17.08)

*TP*  true positive, *TN*  true negative, *FP*  false positive, *FN*  false negative.

**Figure 3:**
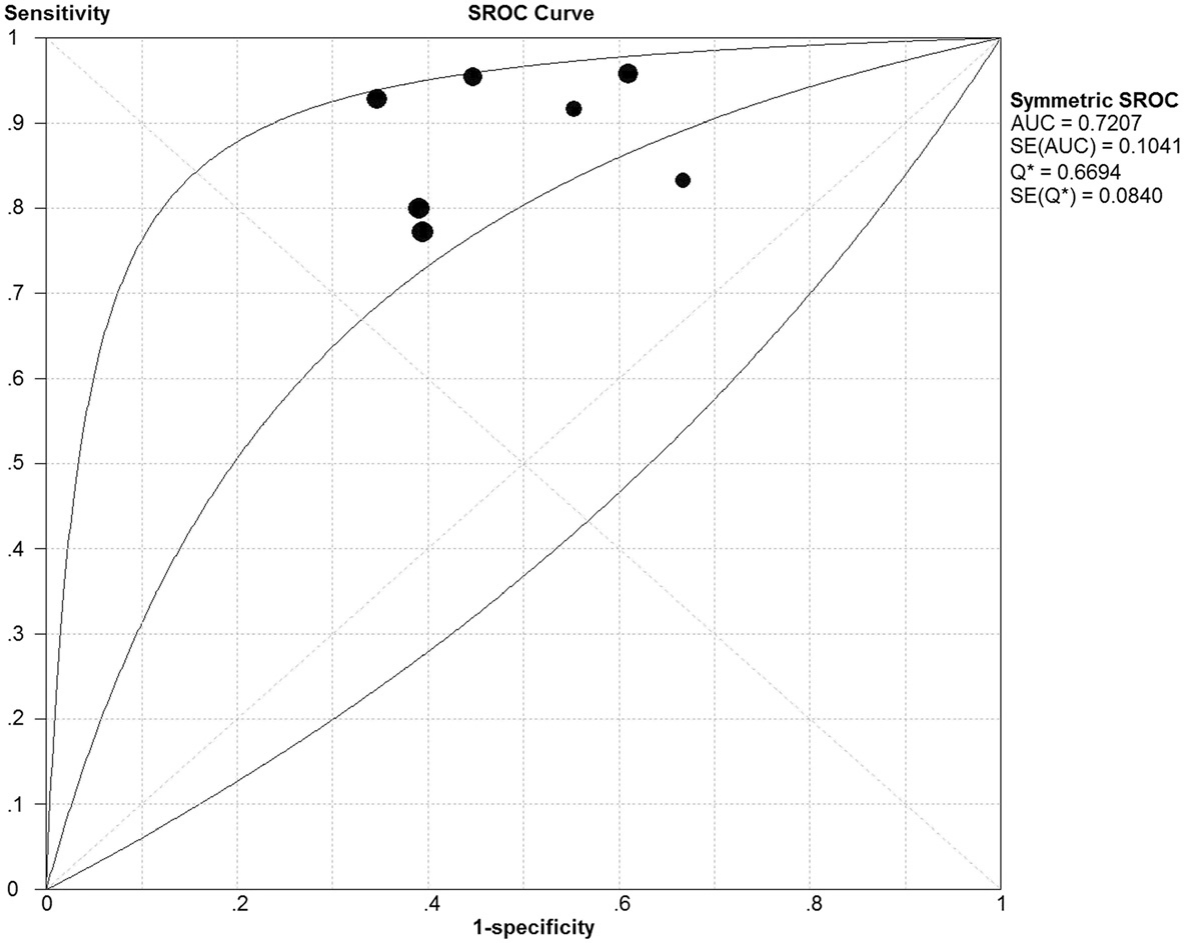
The area under the symmetrical summary receiver operating characteristic curve.

The sROC curve revealed no “shoulder-arm” plot, suggesting no threshold effect. The area [±standard error (±SE)] under the symmetrical sROC curve was 0.7207 ± 0.1041 (Figure 3).

### Malignant lesions

A total number of 70 patients were confirmed to have malignant lesions, with a cancer prevalence of 26.2% (70/267; ranging from 19.6% [15] to 40.0% [13,16]). Among them, 36 (51.4%) lesions were confirmed to be papillary cancers, 16 (22.9%) lesions were follicular cancers, the rest were other types of malignant tumors. ^18^ F-FDG PET or PET/CT correctly detected 62 lesions (88.6%). There were 8 false negative cases: 5 papillary cancers and 3 tumors of uncertain malignant potential (TUMP).

### Benign lesions

197 patients were confirmed to have benign lesions. 107 (54.3%) lesions were diagnosed as adenomas (follicular adenoma: 83, Hürthle cell adenoma: 12 and oxyphilic adenoma: 12), 55 (27.9%) as nodular hyperplasia, 29 (14.7%) as multinodular goiters and the other 6 (3.1%) as thyroiditis. PET or PET/CT only correctly judged 108 lesions (54.8%). There were considerable false positive cases: 48 adenomas (follicular adenoma: 28, Hürthle cell adenoma: 12 and oxyphilic adenoma: 8), 19 nodular hyperplasia, 18 multinodular goiters and 4 thyroiditis.

### The differentiation of malignant and benign lesions

6 studies described age and gender in detail [10-13,15,16]. We found there was no statistical difference of age or sex between malignant and benign lesions (*P* > 0.05). 5 studies analyzed diameters, and the diameter could not be used to differentiate malignant and benign lesions, either (P > 0.05) [10,12,13,15,16]. 4 studies listed SUV in detail [10,12,13,16]. Although SUVmax was higher in malignant lesions (8.6 ± 7.0 vs 5.0 ± 4.3; *P* < 0.01), there was still a great overlap. The best cut-off value of SUVmax for differentiation was 2.05, yet with a high sensitivity of 89.8% and low specificity of 42.0%.

## Discussion

FDG-avid thyroid nodules detected on PET scan are at a high risk of malignancy but the utility of ^18^ F-FDG PET in the presurgical characterization of indeterminate thyroid nodules at cytology is controversial, due to discordant results in the literature [16]. The most recent American Thyroid Association (ATA) guidelines recommend exploring any FDG-avid nodule by FNAB (recommendation A1: Strongly recommends) but do not recommend the routine presurgical use of PET to detect malignancy (recommendation E: Recommends against) [3].

In our meta-analysis, ^18^ F-FDG PET or PET/CT correctly detected 62/70 malignant lesions, with a very high pooled sensitivity of 89.0%. Furthermore, SUV showed valuable in differentiating malignant lesions from benign ones (*P* < 0.01). We considered that PET was a useful tool to predict malignancy.

Vriens et al. has already meta-analyzed the utility of ^18^ F-FDG PET in detecting patient with indeterminate FNAB results [23]. Our pooled sensitivity and specificity were similar to theirs. Compared with their analysis, our study was more up-dated and included more patients in order to enhance our statistical accuracy. Besides, we deleted one study with no FDG negative results [17]; because LR- could not be calculated under this circumstance, and with a specificity of 0%, which would lead to inconsistency between studies.

Previous systemical study concluded that a false negative PET result only occurred in nodules with a greatest histologic dimension <1.5 cm [23]. In our analysis, there were 8 false negative cases, and 5 lesions with a dimension ≥ 2.0 cm. Therefore, we thought the size was not the sufficient reason to explain false negative phenomena. Further studies should be performed to better demonstrate it. Moreover, we noted that the majority of false negative results occurred in one study [16]; but in this study, the investigators regarded TUMP as malignant lesions, we thought it may cause bias and consequently make more FDG false negative cases. We suggested whether these patients had true malignant lesion should be further investigated.

Variable physiologic FDG uptake, and asymmetric FDG distribution in the neck can confound image interpretation. As a consequence, false positive is inevitable [24]. We detected a considerable number of false positive cases in the analysis. The pooled specificity was only 55.0%. Therefore, we still should be very cautious when making a conclusion of malignancy. Yang et al. once demonstrated that the presence of focal uptake with high SUVmax and calcification detected on CT images correlates with a high likelihood of thyroid malignancy [25]. Hence, we considered that sometimes we could utilize CT imaging for reference in order to ameliorate the diagnostic accuracy.

Currently, the cost of PET is relatively high; some studies did not encourage it before thyroid surgery [26,27]. However, there remained other reasons to consider despite this cost-effectiveness. As in larger lesions, the complication rates can be higher (because of extension toward the large vessels, trachea, or recurrent laryngeal nerve), and these patients may benefit most from reassurance of a true-negative PET [23].

According to our analysis, PET correctly diagnosed 63.7% (170/267) of patients with indeterminate FNAB results. Although there were some false positive cases, over half patients (108/197) could avoid unnecessary surgery after PET. We believed that PET was useful in assisting treatment.

However, there are several limitations that are worth mentioning, including publication bias, selector bias, or diagnostic workup bias. For example, different studies used different definition of FDG PET positivity and malignancy. Besides, some studies were evaluated by PET while others were evaluated by PET/CT. Furthermore, the heterogeneity of the population still existed, particularly the vast variation in the prevalence of malignancy in different parts of the world with both endemic goiter areas and iodine-sufficient areas.

## Conclusions

Our comprehensive meta-analysis of the literature revealed that ^18^ F-FDG PET or PET/CT showed a high sensitivity in detecting cancers in patients with indeterminate FNAB results. Further examination, such as hemithyroidectomy, was strongly recommended when an FDG-avid lesion was detected.

## Competing interest

The authors’ declare that they have no competing interest.

## Authors’ contributions

Conception and design: NW and YL, Acquiring data, or analyzing and interpreting data: NW and HZ, Drafting the manuscript: NW, Revising the manuscript and enhancing its intellectual: Y L. All authors’ read and approved the final manuscript.
