# Erratum: Is fluorine-18 fluorodeoxyglucose positron emission tomography useful for the thyroid nodules with indeterminate fine needle aspiration biopsy? a meta-analysis of the literature

**DOI:** 10.1186/s40463-014-0043-5

**Published:** 2014-11-14

**Authors:** Ningjian Wang, Hualing Zhai, Yingli Lu

**Affiliations:** Department of Endocrinology and Metabolism, Shanghai Ninth People’s Hospital, Affiliated to Shanghai Jiaotong University School of Medicine, No. 639, Zhizaoju Road, Shanghai, 200011 China

## Erratum

After publication of this work [1], we noted that we inadvertently failed to fully complete the referencing for the article. The authors would like to apologise for these oversights.

The new, additional references to the sentences can be found in the following sections below:

### Background – first paragraph

It represents approximately 1% of all cancers, corresponding to an incidence of up to 56, 460 new cases per year in the United States, with increasing incidence over the last decades [2]. Early identification and diagnosis is important in appropriate treatment of thyroid cancer, as delays in the diagnosis are associated with increased mortality [3].

### Background – third paragraph

Because only 20% to 30% of these nodules are malignant, most patients are undergoing unnecessary thyroid surgery with the potential risk of irreversible complications [2]. Unfortunately, at present, there is no alternative algorithm for a more conservative management of patients with thyroid nodules of indeterminate cytopathology [3].

### Methods - Study quality – second sentence

This widely used tool consists of 14 items that cover patient spectrum, reference standard, disease progression bias, verification and review bias, clinical review bias, incorporation bias, test execution, study withdrawals, and intermediate results [2].

### Methods - data analysis – second paragraph

Because sensitivity and specificity often are related inversely because of the threshold effect, study heterogeneity in these diagnostic test characteristics was observed using a summary receiver operating characteristic (sROC) curve for which the area under the curve (AUC) was calculated [2].

### Results - study quality – second sentence

Besides, none of the reviewed articles interpreted the 18 F-FDG PET and histology data in combination with other clinical data that would be available in practice (QUADAS Item 12) [2].

### Discussion – first paragraph – second sentence

The most recent American Thyroid Association (ATA) guidelines recommend exploring any FDG-avid nodule by FNAB (recommendation A1: Strongly recommends) but do not recommend the routine presurgical use of PET to detect malignancy (recommendation E: Recommends against) [4].
